# Serotype and Genotype (Multilocus Sequence Type) of Streptococcus suis Isolates from the United States Serve as Predictors of Pathotype

**DOI:** 10.1128/JCM.00377-19

**Published:** 2019-08-26

**Authors:** April A. Estrada, Marcelo Gottschalk, Stephanie Rossow, Aaron Rendahl, Connie Gebhart, Douglas G. Marthaler

**Affiliations:** a Department of Veterinary and Biomedical Sciences, College of Veterinary Medicine, University of Minnesota, St. Paul, Minnesota, USA; b Faculty of Veterinary Medicine, University of Montreal, Saint-Hyacinthe, Quebec, Canada; c Veterinary Diagnostic Laboratory, College of Veterinary Medicine, University of Minnesota, St. Paul, Minnesota, USA; d Veterinary Diagnostic Laboratory, College of Veterinary Medicine, Kansas State University, Manhattan, Kansas, USA; University of Tennessee at Knoxville

**Keywords:** MLST, *Streptococcus suis*, multilocus sequence typing, pathogenic, pathotype, porcine, serotyping

## Abstract

Streptococcus suis is a significant cause of mortality in piglets and growing pigs worldwide. The species contains pathogenic and commensal strains, with pathogenic strains causing meningitis, arthritis, endocarditis, polyserositis, and septicemia. Serotyping and multilocus sequence typing (MLST) are primary methods to differentiate strains, but the information is limited for strains found in the United States. The objective of this study was to characterize the diversity of 208 S. suis isolates collected between 2014 and 2017 across North America (mainly the United States) by serotyping and MLST and to investigate associations between subtype and pathotype classifications (pathogenic, possibly opportunistic, and commensal), based on clinical information and site of isolation. Twenty serotypes were identified, and the predominant serotypes were 1/2 and 7. Fifty-eight sequence types (STs) were identified, and the predominant ST was ST28. Associations among serotypes, STs, and pathotypes were investigated using odds ratio and clustering analyses. Evaluation of serotype and ST with pathotype identified a majority of isolates of serotypes 1, 1/2, 2, 7, 14, and 23 and ST1, ST13, ST25, ST28, ST29, ST94, ST108, ST117, ST225, ST373, ST961, and ST977 as associated with the pathogenic pathotype. Serotypes 21 and 31, ST750, and ST821 were associated with the commensal pathotype, which is composed of isolates from farms with no known history of S. suis-associated disease. Our study demonstrates the use of serotyping and MLST to differentiate pathogenic from commensal isolates and establish links between pathotype and subtype, thus increasing the knowledge about S. suis strains circulating in the United States.

## Abstract (precis)

Streptococcus suis is a significant cause of mortality in piglets and growing pigs worldwide. The species contains pathogenic and commensal strains, with pathogenic strains causing meningitis, arthritis, endocarditis, polyserositis, and septicemia. Serotyping and multilocus sequence typing (MLST) are primary methods to differentiate strains, but the information is limited for strains found in the United States.

## INTRODUCTION

Disease caused by Streptococcus suis is a significant economic and welfare concern in the swine industry. S. suis is a Gram-positive bacterium, and the species contains pathogenic and commensal strains. Pathogenic S. suis strains are associated with meningitis, arthritis, endocarditis, polyserositis, and septicemia in piglets and growing pigs (1, 2), and S. suis strains isolated from neurological or systemic tissues (brain/meninges, joints, and heart) are commonly considered the primary pathogens (2–4). Commensal strains normally reside in the upper respiratory tract of pigs, with pigs commonly serving as carriers (1, 5, 6). S. suis can be an opportunistic pathogen associated with coinfections with other bacterial and viral pathogens (2, 3). In addition, some S. suis strains have zoonotic potential, causing meningitis in humans (7).

Serotyping and multilocus sequence typing (MLST) are commonly used to differentiate S. suis strains, and numerous subtypes exist within the S. suis species. Traditionally, serotyping uses the coagglutination test with reference antisera to subtype S. suis strains, and 35 known S. suis serotypes (1 to 34 and 1/2) exist. Six of those serotypes have been reclassified as Streptococcus orisratti (serotypes 32 and 34), Streptococcus parasuis (serotypes 20, 22, and 26), or Streptococcus ruminantium (serotype 33) (8–10). The development of PCR-based and whole-genome sequencing (WGS) techniques can also be used to serotype and identify strains that were previously nontypeable through traditional serological methods (11, 12).

A 1992 United States study investigated the serotype distribution of S. suis in porcine samples from Minnesota and reported the prevalence of serotypes 2 to 9 and 11, of which serotype 2 was the predominant serotype associated with neurological disease (3). A 1993 U.S. study identified serotypes 1 to 8 and 1/2 in naturally infected pigs primarily from a single state, with serotype 2 being the predominant serotype, followed by serotypes 3, 4, 7, 8, 1, 5, 1/2, and 6 (13). A large U.S. study in 2009 investigated the serotype distribution of S. suis strains collected from 2003 to 2005 from 17 states, illustrating that the distribution of strains was similar to Canada (14). In both countries, serotypes 1/2, 2, 3, 7, and 8 were most prevalent in diseased pigs (14, 15) which is dissimilar to the distribution in Europe, in which serotype 2 occurs at a considerably higher percentage of isolates than in North America (16).

MLST is a nucleotide sequence-based technique for subtyping bacteria, and a standard MLST scheme has been developed for S. suis, with 1,161 registered sequence type (ST) profiles as of 28 February 2019 (17) (pubmlst.org). Global MLST studies of S. suis identified ST1, ST25, and ST28 as the most prevalent STs in swine (18–21). In North America, ST25 and ST28 are more common among strains recovered from diseased animals, while ST1 strains are more prevalent in Europe and Asia (18, 20, 22). However, these studies address MLST for serotype 2 strains and may not apply to the remaining serotypes.

Previously, studies have classified isolates into pathotypes based on clinical information and site of isolation (3, 4). Our objective was to combine information on pathotype with serotype and ST to address the limited information on current S. suis strains circulating within the United States. In total, 208 porcine S. suis isolates from North America were characterized by serotyping and MLST to determine the population and distribution of S. suis in the United States. Furthermore, the serotype and MLST data were used to investigate associations with the pathogenic and commensal pathotypes with the goal to identify pathogenic- and commensal-specific serotype and MLST patterns. Identifying the major disease-causing strains can promote the development of treatment and control plans. Our research seeks to identify pathogenic strains to track isolates in an outbreak, select strains for a vaccine, and develop effective treatment and control plans.

## MATERIALS AND METHODS

### Selection of S. suis isolates.

A total of 208 S. suis isolates were selected for the project. Most of the S. suis isolates were obtained from routine diagnostic cases submitted between April 2014 and July 2017 to the University of Minnesota Veterinary Diagnostic Laboratory (UMNVDL) or the Kansas State Veterinary Diagnostic Lab (KSVDL). Further commensal isolates were collected from 9 different farms with a lack of systemic S. suis clinical disease. Isolates that met our pathotype criteria (defined below) were selected from as many states as possible (*n* = 20) to minimize sample bias and increase geographic diversity to represent the major regions of the U.S. swine industry. S. suis isolates were verified to the species level by matrix-assisted laser desorption ionization–time of flight mass spectrometry (MALDI-TOF MS) (Microflex device, Bruker Daltonics GmbH, Germany) (23).

Multiple isolates may be recovered from healthy pigs due to the native microflora of the upper respiratory tract, while a single isolate is generally responsible for systemic infections (24). To limit the bias in isolating and selecting strains associated with clinical signs, a pathotype category system was developed for the S. suis isolates similar to previously published methods (4, 25). “Pathogenic” isolates were obtained from the brain/meninges, joint, heart, or liver and reported as the primary cause of meningitis, arthritis, epicarditis, or septicemia in diagnostic reports by pathologists. “Possibly opportunistic” isolates were from lung samples submitted to the diagnostic lab from pigs without signs of neurological or systemic disease and included two isolates from nasal samples from farms with a clinical outbreak of S. suis disease. “Commensal” isolates were from laryngeal, tonsil, or nasal samples retrieved from farms with no known history or current control methods for S. suis disease.

### Serotyping, MLST via whole-genome sequencing.

Isolates were recultured for 24 to 48 h at 37°C on blood agar plates (tryptic soy agar [TSA] with 5% sheep blood) (Thermo Fisher Scientific, Waltham, MA, USA) and sent for serotyping to the bacterial serology laboratory at the Diagnostic Service of the Faculty of Veterinary Medicine of the Université de Montréal, Canada. The serotyping was done through the coagglutination test with reference antisera (26–29). Nontypeable samples (samples which failed to react with the serum panel, autoagglutinated, or reacted to several sera) were further serotyped by PCR (30), a technique that cannot differentiate serotype 2 from 1/2 and serotype 1 from 14.

The S. suis DNA was extracted using the protocol for cultured cells from the QIAamp DNA kit (Qiagen Inc., Germantown, MD, USA) and submitted to the University of Minnesota Genomic Center (UMGC, St. Paul, MN, USA) for library preparation using Nexture TX (Illumina, San Diego, CA), and next-generation sequencing was performed on a HiSeq 2500 instrument (Illumina) with 250-bp paired-end reads. Illumina sequencing reads for each isolate were processed using Trimmomatic (31) with an average quality cutoff of 20 (2.3 million average reads per sample). Strains were again confirmed as S. suis by having a 96.6% to 100% nucleotide identity to the 1,662-bp S. suis-specific recombination/repair protein (*recN*) sequence (Streptococcus suis 05HAS68, GenBank accession number CP002007) using the S. suis serotyping pipeline (32).

The isolates with serotypes 2 or 1/2, 1 or 14, or lacking a subtype using traditional serotyping or PCR methods were serotyped using the WGS data and the S. suis serotyping pipeline (https://github.com/streplab/SsuisSerotyping_pipeline) (12). The pipeline uses the *cpsK* gene (Streptococcus suis NSUI002, GenBank accession number CP011419) missense mutation at position 161 to differentiate serotypes 2 and 1/2 and serotypes 1 and 14. Isolates undifferentiable by WGS were categorized as serotype “1or14” or NT (nontypeable) in the downstream analysis.

*In silico* MLST analysis was performed using the Short Read Sequence Typing for Bacterial Pathogens (SRST2) program (http://katholt.github.io/srst2), which maps reads to MLST references (33). The ST allele sequences and profiles were obtained from the S. suis MLST database (https://pubmlst.org/ssuis/) (34). Novel ST allele sequences were confirmed by PCR amplification and Sanger sequencing of the *aroA*, *cpn60*, *dpr*, *gki*, *mutS*, *recA*, or *thrA* genes (17). The primers used for the amplification and sequencing of the *mutS* gene were mutS forward (5′-AAGCAGGCAGTCGGCGTGGT-3′) and mutS reverse (5′-AGTACAAACTACCATGCTTC-3′) as described (35). STs were grouped into major clonal complexes (CCs) using the entire MLST database and the eBURST software (36). Groups were defined with the strict parameters for determining single-locus variants (match of 6 or more loci). The entire S. suis MLST database was displayed as a single eBURST diagram by setting the group definition to zero of seven shared alleles.

### MLST clustering analysis.

Alignments, sequence identity calculations, and construction of the MLST sequence identity heatmap for basic clustering analysis were performed with R software (v.3.4.3) (37) and R packages (38–41). The concatenated sequences of the seven MLST alleles were aligned with MUSCLE (v.3.8.31) (42), and sequence identities were calculated. The sequence identity scores were used to generate a heatmap based on Euclidian distances and neighbor joining clustering.

### Statistical analysis.

Basic data transformation and plotting for statistical analyses were performed using R software and R packages (43–45). Ternary plots of subtypes and pathotypes were generated using the R package Ternary (v.1.0.2) (46). The pathotype boundaries were assigned and color-coded using 50% as a cutoff. Odds ratio (OR) analysis was used to test all pathotype-subtype combinations containing more than a single isolate, and 95% confidence intervals (CIs) were generated using Fisher’s exact test. For each combination, the 2 by 2 table was created comparing that pathotype and subtype against all others. Similar 2 by 2 tables were generated for testing pathotype and serotype-ST-combinations by chi-square and Fisher’s exact tests. ORs greater than 1 with a 0.3 minimum lower limit were considered biologically significant. The minimum lower limit of 0.3 was calculated as the average lower limit among the combinations, is specific to our data set, and was selected for the identification of biologically meaningful relationships. An infinite (Inf) OR for a pathotype-subtype combination refers to a subtype that occurred in only one pathotype. The associations within and between types were investigated using multiple correspondence analysis (MCA), with the FactoMineR (v.1.41) and factoextra (v.1.0.5) packages (47, 48), by setting the serotype, ST, and pathotype as the three variables.

### Data availability.

The reads associated with the samples were deposited in the NCBI Sequence Read Archive under accession numbers SRR9123061 to SRR9123268 (see Table S1).

## RESULTS

### Serotype and ST distributions of S. suis in the United States. Characterization of S. suis isolates by serotyping and MLST.

**FIG 1:**
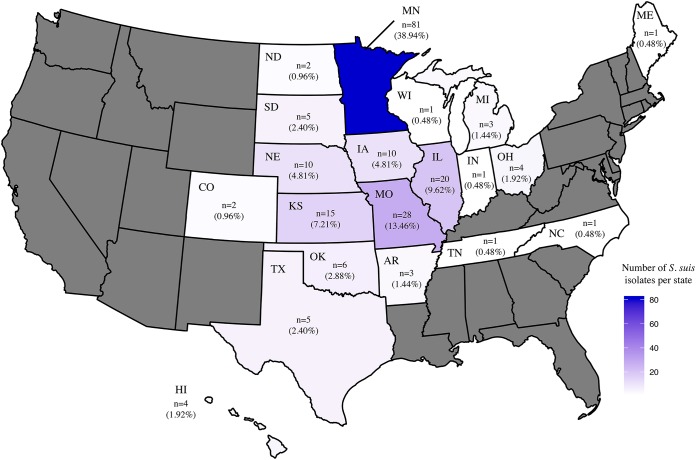
The number and percentage of S. suis isolates characterized from each state. States are colored according to the number of isolates characterized from each state. States without isolates are gray. Isolates from Canada (*n* = 4) and Mexico (*n* = 1) are not shown.

A total of 208 S. suis isolates were characterized, of which 203 were from the United States, 4 from Canada, and 1 from Mexico (Fig. 1). The clinical history and tissue of origin of the isolates were used to determine the pathotype, and the 208 isolates were classified as pathogenic (*n* = 139), possibly opportunistic (*n* = 47), and commensal (*n* = 22) (Table 1). The *recN* segment from S. suis was identified in the whole-genome sequences of all the 208 strains (>99% coverage of the gene and 40× to 314× depth), indicating that the isolates were S. suis.

**TABLE 1:** Distribution of S. suis isolates by pathotype classification and tissue of origin

Pathotype	Tissue of origin	*n*
Pathogenic (*n* = 139, 66.8%)	Brain/meninges/spinal cord	56
	Heart	25
	Joint/synovial fluid	23
	Liver	35
Possibly opportunistic (*n* = 47, 22.6%)	Lung	45
	Nasal	2
Commensal (*n* = 22, 10.6%)	Laryngeal	1
	Nasal	17
	Tonsil	4

Serotyping identified 20 different serotypes representing 94.2% of the strains, while 5.3% were nontypeable (indicated as NT) and 0.5% could not be differentiated between serotype 1 and 14 (*n* = 1) by coagglutination, PCR, or WGS (see Table S2 in the supplemental material). The predominant serotypes were 1/2 (*n* = 54) and 7 (*n* = 23).

*In silico* MLST analyses were performed on the WGS data, and the samples had an average depth of 155× across the seven loci. STs could not be determined for four isolates because one housekeeping gene necessary for MLST classification was not identified in these isolates (referred to as NF, see Table S1 in the supplemental material). Fifty-eight different STs were identified for the remaining 204 isolates, indicating high diversity among the isolates (see Table S3 in the supplemental material). Twenty of these STs were previously defined, while 38 were newly identified (961 to 969, 971 to 998, and 1001; *n* = 56). The predominant ST was ST28 (*n* = 52), followed by ST94 (*n* = 18), ST1 and ST108 (*n* = 17 each).

### Relationship between serotypes and STs.

**FIG 2:**
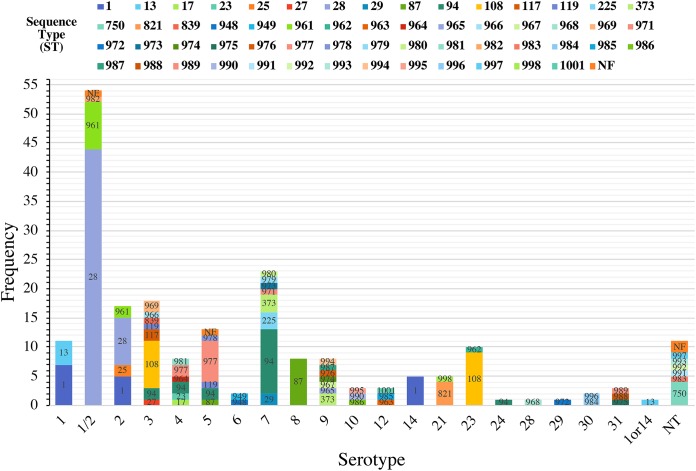
Distribution of S. suis STs by serotype. The stacked histogram illustrates the serotypes identified in this study, which were subdivided by STs. The *x* axis represents each serotype while the *y* axis represents the frequency of each serotype. Bar sections are labeled with their respective STs. The category 1or14 and (nontypeable (NT) represents isolates with serotypes that could not be differentiated by coagglutination, PCR, or WGS.

The distribution of STs by serotype illustrated the diversity of the S. suis strains (Fig. 2). Fifteen of the 20 serotypes identified contained multiple STs, with the number of different STs within a single serotype ranging from 2 to 8. The predominant serotype 1/2 contained three STs (ST28 [*n* = 44], ST961 [*n* = 8], and ST982 [*n* = 1]). Serotypes 8, 14, 24, 28, and 29 contained a single ST each, namely, ST87, ST1, ST94, ST968, and ST972, respectively. However, serotypes 24, 28, 29, and 1or14 contained only a single isolate.

### Distribution of pathotypes by serotype and ST.

**FIG 3:**
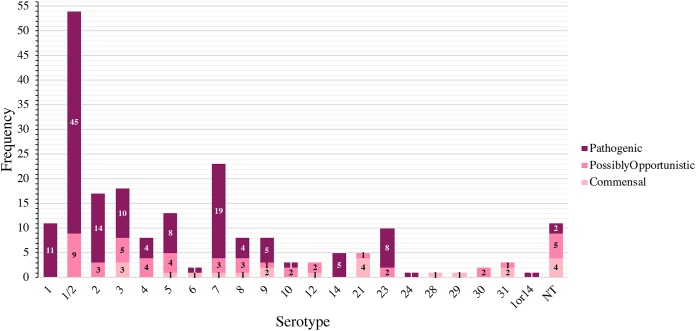
Distribution of S. suis pathotypes by serotype. The stacked histogram illustrates the serotypes identified in this study, which were subdivided by pathotype (pathogenic, possibly opportunistic, and commensal). The *x* axis represents each serotype while the *y* axis represents the frequency of each pathotype. Bar sections are labeled with their respective pathotypes. The category 1or14 and NT (nontypeable) represents isolates with serotypes that could not be differentiated by coagglutination, PCR, or WGS.

**FIG 4:**
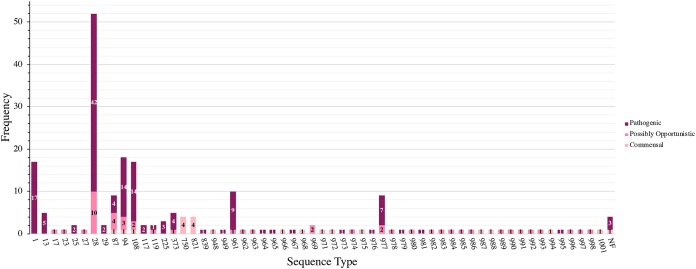
Distribution of S. suis pathotypes by ST. The stacked histogram illustrates the STs identified in this study, which were subdivided by pathotype (pathogenic, possibly opportunistic, and commensal). The *x* axis represents each ST while the *y* axis represents the frequency of each pathotype. Bar sections are labeled with their respective pathotypes. Not found (NF) indicates ST could not be determined because one housekeeping gene could not be identified for MLST classification.

The distribution of S. suis pathotypes by serotype is shown in Fig. 3. Serotype 1/2 contained the most pathogenic isolates (*n* = 45/54), followed by serotypes 7 (*n* = 19/23), 2 (*n* = 14/17), and 1 (*n* = 11/11). Serotypes 1 and serotype 14 (*n* = 5) were composed entirely of pathogenic isolates. The distribution of pathotypes by ST is shown in Fig. 4. ST28 contained the most pathogenic isolates (*n* = 42/52), followed by ST1 (*n* = 17/17), ST94 (*n* = 14/18), and ST108 (*n* = 14/17), of which only ST1 was composed entirely of pathogenic isolates. Additional pathogenic STs included ST961 (*n* = 9/10), ST977 (*n* = 7/9), and ST13 (*n* = 5/5). Twelve STs contained isolates only classified as commensal, with ST750 and ST821 containing more than a single commensal isolate.

### Associations among pathotypes, serotypes, and STs by analysis of proportions and OR. Associations between pathotype and serotype.

**FIG 5:**
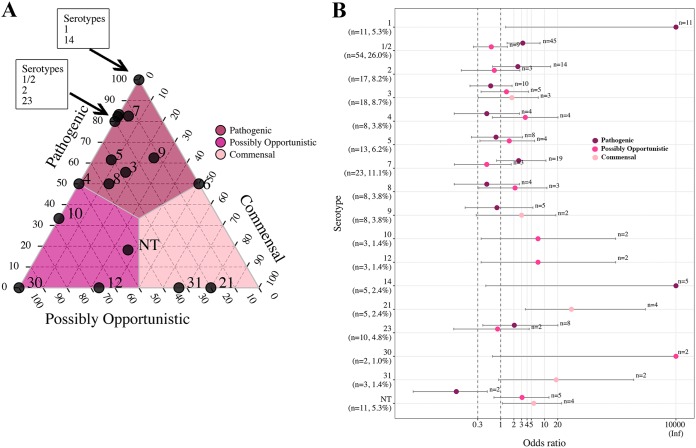
Ternary and OR plots summarizing the associations between S. suis pathotype and serotype. Only serotypes containing more than a single isolate are illustrated in the ternary plot, and only pathotype-serotype combinations containing more than a single isolate are illustrated in the OR plot. (A) The proportions of isolates classified as the pathogenic, possibly opportunistic, and commensal pathotype in each of the 16 serotypes (and the NT category) were plotted. The gray lines and color shading denote pathotype boundaries. (B) OR plot for 16 serotypes (and the NT category) versus pathotype. The dotted lines illustrate the minimum lower limit (OR, 0.3) and typical threshold (OR, 1) for identifying significant ORs. Error bars represent the 95% confidence intervals. Inf, Infinite. Nontypeable (NT) represents isolates which could not be serotyped using coagglutination, PCR, or WGS.

Proportions and OR analyses were used to investigate pathotype associations with serotype for serotypes (proportions) or serotype-pathotype combinations (OR analysis) that contained more than one isolate. Between 80% and 100% of serotypes 1, 1/2, 2, 7, 14, and 23 were classified as the pathogenic pathotype (Fig. 5A), and these associations were supported by OR analysis (Fig. 5B). In the ternary plot, serotypes 3, 5, and 9 demonstrated a moderate association with the pathogenic pathotype, with 56% to 63% of isolates classified as pathogenic. However, the association between pathotype and serotype was not supported by OR analysis. OR analysis supported associations of serotypes 10 and 12 with the possibly opportunistic pathotype, with 67% of isolates classified as possibly opportunistic in the ternary plot. Serotypes 21 and 31, with 67% to 80% of isolates classified as commensal in the ternary plot, were supported as commensal pathotypes by OR analysis.

### Associations between pathotype and ST.

**FIG 6:**
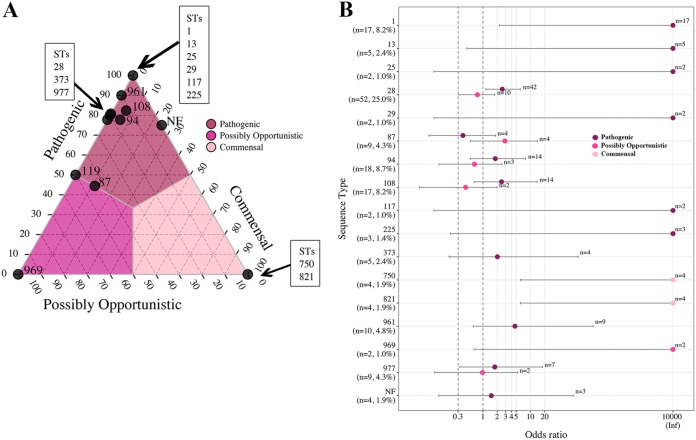
Ternary and OR plots summarizing the associations between S. suis pathotype and ST. Only STs containing more than a single isolate are illustrated in the ternary plot, and only pathotype-ST combinations containing more than a single isolate are illustrated in the OR plot. (A) The proportions of isolates classified as the pathogenic, possibly opportunistic, and commensal pathotype in each of the 17 STs (and the NF category) were plotted. The gray lines and color shading denote pathotype boundaries. (B) OR plot for 16 STs (and the NF category) versus pathotype. ST119 contained a single isolate of each pathogenetic and possibly opportunistic pathotype failing to meet the criteria for the plot. The dotted lines illustrate the minimum lower limit (OR, 0.3) and typical threshold (OR, 1) for identifying significant ORs. Error bars represent the 95% confidence intervals. Infinite (Inf) is represented by the value 10,000 for visualization purposes. Not found (NF) indicates ST could not be determined because one housekeeping gene could not be identified for MLST classification.

Proportions and OR analysis were used to investigate pathotype associations with ST for STs (proportions) or ST-pathotype combinations (OR analysis) that contained more than one isolate. The ternary plot of the 58 STs (and the NF category) illustrated a clear differentiation by pathotype for all STs except ST87 and ST119 (approximately 50% pathogenic) (Fig. 6A). Twelve STs and the NF category contained over 75% of isolates classified as pathogenic, including ST1, ST13, ST25, ST28, ST29, ST94, ST108, ST117, ST225, ST373, ST961, and ST977, which demonstrated the same associations by OR (Fig. 6B). ST969 had an association with the possibly opportunistic pathotype, which was supported by OR. The commensal pathotype demonstrated a strong association with ST750 and ST821, which was supported by OR analysis.

### Odds ratio and MCA of pathotypes, serotypes, and STs.

**FIG 7:**
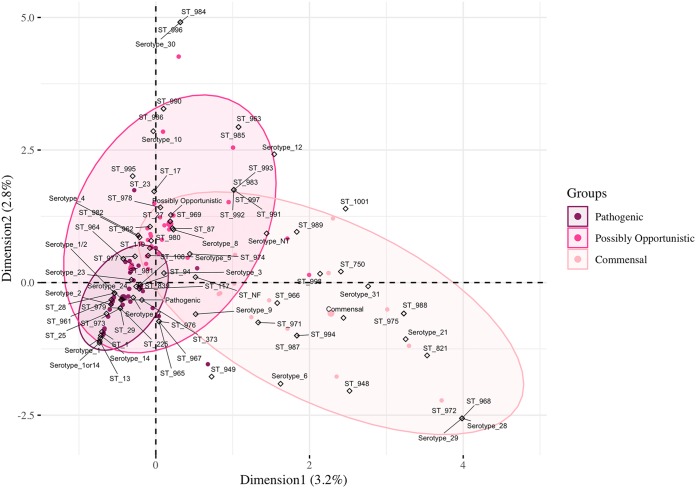
Three-way MCA analyzing the associations among pathotypes, serotypes, and STs. The ellipses represent 95% of isolates in each pathotype. Dots represent isolates colored by their respective pathotype, and hollow diamonds represent the three active variables (pathotype, serotype, and ST). Nontypeable (NT) represents isolates which could not be serotyped using coagglutination, PCR, or WGS. Not found (NF) indicates ST could not be determined because one housekeeping gene could not be identified for MLST classification.

Initially, OR was used to investigate the relationships between pathotype and serotype-ST-combinations, but significance relationships were lacking for the combinations (OR data not shown). Then, MCA was performed to analyze the possible relationships among all serotypes, STs, and pathotypes (Fig. 7). The first and second dimensions of the analysis only represent 6% of the data. The ellipses represent 95% of isolates in each pathotype. All the subtypes demonstrating a strong association with the pathogenic pathotype by OR analysis (Fig. 5 and 6) fell within the overlapping 95% ellipses for multiple pathotypes by MCA (Fig. 7). Five serotypes and 13 STs in the commensal pathotype lacked overlapping ellipses. Serotypes 21 and 31 lacked any isolates with the pathogenic pathotype (Fig. 3), while ST750 and ST821 contained only isolates with the commensal pathotype (Fig. 4). The limited representations of the MCA data (6% variance) and the overlapping ellipses indicate a lack of relationship between serotype, ST, and pathotype, highlighting potential confounding factors for predicting pathogenic isolates based on both serotyping and MLST together. Thus, the relationship between pathotype, serotype, and ST is lacking for the pathogenic and possibly opportunistic pathotypes.

### Associations between pathotype and MLST CC by analysis of proportions and OR. Identification of S. suis CCs.

**FIG 8:**
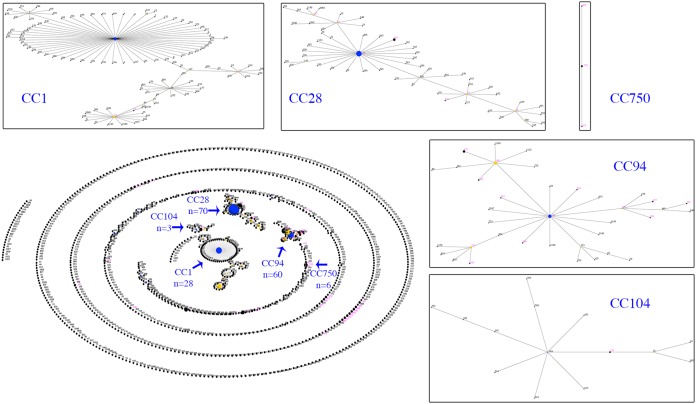
eBURST illustration of the global S. suis population. Primary founders (blue) are positioned at the center of the cluster and subgroup founders are shown in yellow. Clusters of linked STs correspond to CCs. Magenta dots mark the STs identified in our study and arrows mark the CCs relevant to this study. Individual CCs have been expanded to illustrate relationships among STs.

To investigate the population structure of our S. suis isolates by MLST, the STs were assigned into CCs defined by eBURST, using the entire S. suis MLST database and our 58 STs (Fig. 8 and Table S3). Using the stringent definition (six of seven shared alleles) for defining a CC, five CCs (CC1, CC28, CC94, CC104, and CC750) with a primary founder were identified from our set of STs. However, multiple STs (*n* = 30) did not form a CC or formed a CC without a primary founder (Table 2). The most diverse CC (CC94) contained isolates from 13 of the 28 STs assigned into CC, compared with CC1, CC28, CC104, and CC750, which contained isolates from 4, 7, 1, and 3 STs, respectively.

**TABLE 2:** Primary S. suis CCs identified in this study

No. of CCs	Total no. of isolates^*a*^	No. of pathogenic isolates	No. of possibly opportunistic isolates	No. of commensal isolates	ST(s)
1	28	21	6	1	1, 17, 23, 87
28	70	58	12	0	25, 27–29, 117, 961^*b*^, 973^*b*^
94	60	43	14	3	94, 108, 119, 373, 839, 962^*b*^, 964^*b*^, 966^*b*^, 969^*b*^, 977^*b*^, 980–982^*b*^
104	3	3	0	0	225
750	6	0	1	5	750, 972^*b*^, 992^*b*^
noCC^*c*^	37	11	14	12	13, 821, 948, 949, 963^*b*^, 965^*b*^, 967^*b*^, 968^*b*^, 971^*b*^, 974–976^*b*^, 978^*b*^, 979^*b*^, 983–991^*b*^, 993–998^*b*^, 1001^*b*^

a STs could not be determined for 4 of the 208 isolates because 1 housekeeping gene could not be identified for MLST classification.

b Novel ST(s).

c CCs were lacking for STs that occurred as singletons or had no determined founder.

### Associations between pathotype and CC.

**FIG 9:**
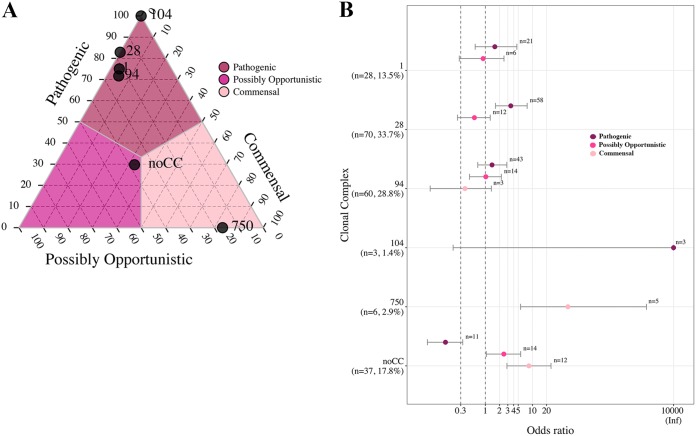
Ternary and OR plots summarizing the associations between pathotype and CC. (A) The proportions of isolates classified as the pathogenic, possibly opportunistic, and commensal pathotype in each of the five CCs (and the noCC category) were plotted. The gray lines and color shading denote pathotype boundaries. (B) OR plot for all five CCs (and the noCC category) versus pathotype. The dotted lines illustrate the minimum lower limit (OR, 0.3) and typical threshold (OR, 1) for identifying significant ORs. Error bars represent the 95% confidence intervals. Infinite (Inf) is represented by the value 10,000 for visualization purposes. noCC represents the group of isolates lacking a CC.

Patterns between CC and pathotype were investigated by proportions and OR analysis. CC1, CC28, CC94, and CC104 were associated with the pathogenic pathotype, and the association was supported by OR analysis (Fig. 9). CC750 was associated with the commensal pathotype and was supported by OR analysis, with 83% of isolates classified as the commensal pathotype. The STs among the group of isolates lacking a CC did not associate with any pathotype.

### Associations among pathotypes, serotypes, and MLST by clustering analysis.

**FIG 10:**
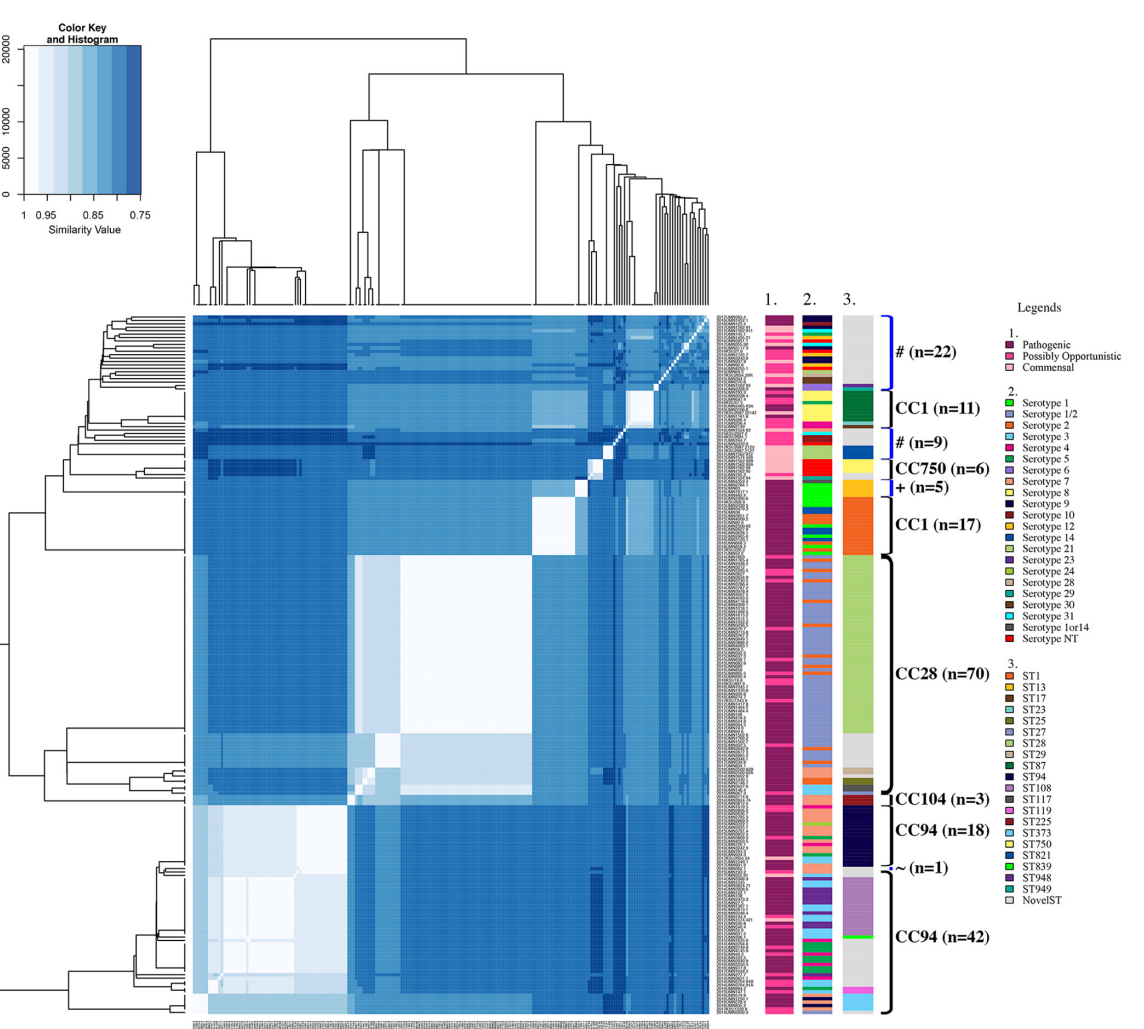
MLST sequence identity heatmap of S. suis. Isolates are annotated (colored rectangles) by pathotype (1), serotype (2), and ST (3). The five CCs are indicated by black brackets, with the number of isolates in the CC. Blue brackets represent clusters of isolates without a CC. Nontypeable (NT) represents isolates which could not be serotyped using coagglutination, PCR, or WGS. #, group of isolates lacking a CC; +, ST13 not within a CC but closest to CC1; ∼, ST979 not within a CC but closest to CC94.

To investigate genetic relationships among the samples and possible associations among serotype, genotype, and pathotype classifications, the MLST allelic sequences were clustered, illustrating 75% to 100% nucleotide sequence identity (Fig. 10). The five main CCs were identifiable in the MLST sequence identity heatmap as clusters of genetically similar strains. CC28 contained the most isolates with the pathogenic pathotype (*n* = 58/70) (Table 2) and was mostly composed of ST28 (predominant ST, *n* = 52) belonging to serotype 1/2 (predominant serotype, *n* = 44) and serotype 2 (*n* = 8) (Fig. 10). CC28 also contained ST961 (*n* = 10) belonging to serotype 1/2 (*n* = 8) and serotype 2 (*n* = 2). MLST clustering analysis demonstrated clustering of CC28 and CC104, and the latter consisted of only three isolates with the pathogenic pathotype (serotype 7, ST225).

CC1 was divided into two groups and clustered with CC750 and isolates without a CC. The first cluster of CC1 contained a concentration of isolates in the pathogenic pathotype (*n* = 17/28), while the second cluster contained 4 pathogenic isolates, 6 possibly opportunistic isolates, and a single isolate with the commensal pathotype (Table 2). The isolates within the first cluster of CC1 were predominantly characterized as ST1 (serotype 1 [*n* = 7/17], serotype 2 [*n* = 5/17], and serotype 14 [*n* = 5/17]). Lacking a CC, the ST13 isolates (*n* = 5; serotype 1 [*n* = 4] and serotype 1or14 [*n* = 1]) clustered with CC1 isolates, demonstrating a possible genetic relatedness to isolates of CC1 and the pathogenic pathotype. Serotypes 1, 2, and 14 and ST1 and ST13 were also associated with isolates of the pathogenic pathotype by proportions and OR. Inversely, CC750 (*n* = 6) consisted of isolates with the commensal (*n* = 5) and possibly opportunistic (*n* = 1) pathotypes and was predominantly composed of isolates characterized as nontypeable (*n* = 5/6) and ST750 (*n* = 4/6). Interestingly, CC750 was closely related to the group of isolates lacking a CC (*n* = 31), which consisted of isolates with the commensal pathotype (*n* = 12/31, multiple serotypes and novel STs), providing further evidence for the association between CC750 and the commensal pathotype.

CC94 was predominantly composed of isolates with the pathogenic pathotype (*n* = 43/60) but contained isolates with all three pathotypes (Table 2). The isolates within CC94 with the pathogenic pathotype were predominantly characterized as ST94 (*n* = 14/43; serotype 7 [*n* = 10/14]), ST108 (*n* = 14/43; serotype 23 [*n* = 8/14]), and ST977 (*n* = 7/43; serotype 5 [*n* = 6/7]). Serotype 7 was the second-most predominant serotype and was associated with the pathogenic pathotype by proportions and OR. Clustering analysis identified CC1, CC28, CC104, and a subset of CC94 as corresponding to the pathogenic pathotype. Isolates within these CCs were predominantly characterized as serotypes 1, 1/2, 2, 7, 14, and 23 and ST1, ST13, ST28, ST94, ST108, ST961, and ST977, providing further evidence of these subtypes corresponding to the pathogenic pathotype.

## DISCUSSION

S. suis is an important swine pathogen, often resulting in neurological and systemic disease caused by pathogenic strains. However, much is still unknown about the population structure of S. suis in the United States. In this study, we utilized serological and molecular typing techniques to investigate the serotype and ST distributions of U.S. isolates. Fourteen of the 20 S. suis serotypes identified in this study were recovered from pigs with clinical disease (*n* = 139). The predominant pathogenic serotypes identified in this study were 1/2 (*n* = 45), 7 (*n* = 19), and 2 (*n* = 14), which have been previously identified as the predominant serotypes from diseased pigs in North America (14, 15, 49, 50). While serotypes 2 and 3 are considered predominant pathogenic serotypes in North America, only 10.6% of the strains in our study were recovered from diseased pigs. Furthermore, the serotype distribution from our study differed from European studies, in which serotypes 2 and 9 are predominant (50, 51). The higher prevalence of serotype 1/2 in North America could be due to a common evolutionary lineage with serotype 2. Genetic analysis by PCR-based serotyping of the *cps* loci demonstrated serotypes 1/2 and 2 share the same genetic profile and cannot be differentiated by serotype-specific *cps* loci (11, 12). Sequencing of the *cpsK* gene reveals a missense mutation permitting the differentiation of serotypes 2 and 1/2 (12), but a PCR protocol has not been implemented yet to differentiate these serotypes.

In our study, the geographic distribution of S. suis was from 20 different states (Table S1), which represent the major swine-producing states in the United States. Variability in the serotype distribution of S. suis has been reported within the same country, which is likely due to natural differences in geographic distribution (13). Geographic distribution of the S. suis serotypes in our study identified serotype 1/2 in 13 of the 20 states, with a concentration in 5 of the 20 states, possibly displaying a geographic distribution pattern of serotype 1/2 in the United States. Serotype 1/2 is also a frequent serotype found in Canada, although at lower levels than serotypes 2 and 3 (52). This prevalence of serotype 1/2 in Canada may contribute to the U.S. serotype distribution through the transport of pigs between the two countries (50). Transport of livestock has been associated with geographic invasion or the emergence of a pathogen in a novel geographic area (53–55). While most transport of pigs to the United States head to harvest facilities, new breeding stock of pigs could be colonialized with new S. suis strains, which could result in the spread of new strains to downstream swine farms. Whole-genome analysis of the U.S. and Canadian serotype 1/2 strains would further clarify the relationship between U.S. and Canadian 1/2 strains.

We anticipated identifying a large number of novel ST profiles due to the inclusion of commensal and possibly opportunistic samples, which are not generally subjected to subtyping by MLST. As a result of this study, 38 novel ST profiles were submitted to the S. suis MLST database. Of the 58 STs identified here, 24 STs were isolated from pigs with clinical disease, and the predominant STs were ST28 (*n* = 42), followed by ST1 (*n* = 17), ST94 (*n* = 14), and ST108 (*n* = 14). In a previous Canadian study in 2011, ST25 was the predominant ST found in Canada, while ST28 was the predominant ST found in the United States (22). Our results confirm ST28 as a predominant pathogenic pathotype, while ST25 represents only 1% of the strains recovered from diseased pigs (*n* = 2). The reason for this low percentage of ST25 isolates in the United States is unclear, and updated ST analysis of S. suis strains from Canada is needed to confirm ST25 as the predominant ST in that country. Our ST distribution also differs from that of European and Asian countries in which ST1 strains, largely characterized as serotype 2, are predominant in diseased pigs (50, 56).

Proportions, OR, and clustering analysis illustrated potential relationships among pathotypes, serotypes, and STs. While multiple pathogenic serotypes and STs were identified in our study, this discussion focuses on serotype and STs with more than four isolates in the pathogenic pathotype. Serotypes 1, 1/2, 2, 7, 14, and 23 as well as ST1, ST13, ST28, ST94, ST108, ST961, and ST977 were frequently identified as pathogenic strains. Based on our pathotype classifications, isolates characterized as pathogenic were linked to neurological or systemic disease, and our analyses provide evidence that these subtypes are potential indicators of virulence. As discussed previously, serotypes 2 and 1/2 are predominant serotypes identified from diseased pigs in North America, supporting our observations of these serotypes as pathogenic strains by proportions, OR, and clustering analysis (14, 15, 49, 50, 52).

Serotypes 1 and 7 are more prevalent in diseased pigs in some European countries than in North America, and pathogenic serotype 1 strains have been linked to the production of muramidase-released protein (MRP), extracellular-factor protein (EF), and suilysin (SLY). Pathogenic serotype 1 strains have been characterized as producing both MRP and EF, with variable production of SLY (16, 18). In one study (18), four of the six serotype 1 strains were MRP^+^EF^+^SLY^+^ and five of the six were either ST1 or ST13, indicating a correlation between serotype 1, ST1, ST13, and virulence. Interestingly, the serotype 1 isolates in the current study were either ST1 (*n* = 7/11) or ST13 (*n* = 4/11) and were associated with the pathogenic pathotype, supporting the previous study. Serotype 7 was the second-most common serotype identified in this study, and 19/23 isolates were characterized as the pathogenic pathotype. Virulence studies on serotype 7 strains demonstrating clinical disease in pigs are limited, but a previous *in vivo* study associated serotype 7 with septicemia and arthritis, with rare cases of meningitis (57). These findings support the classification of serotype 7 as pathogenic.

This study demonstrates that ST appears to be a stronger predictor of pathotype than serotype. While experimental mouse models have demonstrated the virulence of serotype 2 ST1, ST25, and ST28 (22, 56), our analyses also illustrated ST1, ST13, ST28, ST94, ST108, ST961, and ST977 (of various serotypes) as pathogenic. As mentioned previously, we hypothesize that Canadian and U.S. serotype 2 and serotype 1/2 strains share a evolutionary lineage. If so, the observed virulence of serotype 2 ST28 in previous studies may support the virulence of serotype 1/2 ST28, as predicted in our study. Whole-genome single nucleotide polymorphism (SNP)-based phylogenetic analysis of S. suis serotype 2 ST28 strains revealed a unique clade composed of virulent strains capable of inducing severe disease in a murine infection model (58). These strains demonstrated differences in virulence to reference serotype 2 ST28 strains of low virulence. Recently, a study characterized pathogenic Australian serotype 1/2 ST1 strains by core genome single nucleotide polymorphisms and linked the genetic similarity to pathogenic serotype 1/2 ST1 strains from the United Kingdom and Vietnam (59). Our clustering analysis indicates that ST1, ST13, ST94, ST108, ST961, and ST977 may also be pathogenic. It would be of interest to further investigate the virulence properties of serotype 1/2 ST28, as well as ST1, ST13, ST94, ST108, ST961, and ST977 strains isolated in the United States.

In addition to strains in CC1, CC28, and CC104, serotype 9 strains belonging to CC16 (previously CC87) have been isolated from pigs with invasive disease (20). However, the low percentage of serotype 9 strains in our study is reasonable because serotype 9 is predominant in diseased pigs from the Netherlands (16). The serotype 9 strains in this study belong to multiple CCs or occur as singletons and did not demonstrate associations with pathotype. Serotype 9 isolates from diseased and healthy pigs in China were characterized into multiple STs and demonstrated high diversity among the isolates (60). The majority of these serotype 9 isolate STs occurred as singletons and did not form major clonal complexes.

Inversely, commensal S. suis serotypes 21 and 31 and ST750 and ST821 were identified by proportions, OR, and cluster analysis. Studies on S. suis from North America have observed a prevalence of serotype 21 from healthy pigs (26, 27). However, previous studies have identified a limited number of serotype 31 strains from pigs with typical clinical signs of S. suis disease (49, 52, 61, 62). The association between serotype 31 and pathotype remains unclear and requires further investigation.

Associations among serotypes, STs, and pathotypes, although identified by individual analyses, were not evident in the MCA, indicating both serotype and ST together could not indicate pathotype. We investigated additional approaches, such as chi-square and Fisher’s exact tests, but these tests failed to generate significant relationships between both serotype and ST. In addition, we investigated associations between serotype-ST combinations and pathotype by chi-square and Fisher’s exact tests and did not identify any significant associations. One possible explanation for this is the lack of discrimination due to the limitations of sample size within each subtype. Traditional chi-square and Fisher’s exact tests work best on nonsparse data (few zero values) (63, 64). These tests have been used to identify associations between S. suis subtypes and characteristics of pathogenicity. However, most studies involved a limited number of subtypes of interest, while our study focused on all serotypes and STs identified in our sample set. Due to the diversity of the S. suis strains in this study and the large number of subtypes evaluated, the division of our data by pathotype resulted in sparse data. Thus, sparse data limits our ability to conduct certain analyses using common approaches for S. suis. An OR formula was used to evaluate statistical significance of subtype with pathotype, as well as the size of the possible effect, to limit the misidentification of associations due to sample size. For this reason, proportions were used for basic identification of relationships and OR analysis was used for further discrimination of strains.

In summary, our study increases the knowledge on S. suis strains circulating in the United States between 2014 and 2017 by investigating serotype and ST distributions. We identified a diverse set of strains, predominantly serotypes 1/2, 3, and 7, and as ST1, ST28, and ST94. Further investigation by pathotype classification (defined in this study) identified STs that could be differentiated as pathogenic or commensal pathotypes. The predominance of serotype 1/2 strains from clinically affected pigs in our study stresses the importance of expanding studies of virulence traits to other serotypes and STs of S. suis. These findings can be applied to improve the prevention and control of S. suis by selecting strains for diagnostics and vaccine development.

## Supplementary Material

Supplemental file 1

Supplemental file 2

Supplemental file 3
